# Cost-effectiveness of percutaneous coronary intervention versus medical therapy in patients with acute myocardial infarction: real-world and lifetime-horizon data from Taiwan

**DOI:** 10.1038/s41598-021-84853-y

**Published:** 2021-03-10

**Authors:** Chia-Te Liao, Tung-Han Hsieh, Chia-Yin Shih, Ping-Yen Liu, Jung-Der Wang

**Affiliations:** 1 Division of Cardiology, Department of Internal Medicine, Chi-Mei Medical Center, Tainan, Taiwan; 2 Department of Public Health, College of Medicine, National Cheng Kung University, No. 1, University Road, Tainan, 701 Taiwan; 3 Department of Electrical Engineer, Southern Taiwan University of Science and Technology, Tainan, Taiwan; 4 Division of Cardiology, Department of Internal Medicine, National Cheng Kung University Hospital, Tainan, Taiwan; 5 Department of Internal Medicine and Occupational and Environmental Medicine, National Cheng Kung University Hospital Tainan, No. 1, University Road, Tainan, 701 Taiwan

**Keywords:** Health care, Health care economics

## Abstract

Although some studies have assessed the cost-effectiveness of percutaneous coronary intervention (PCI) in acute myocardial infarction (AMI), there has been a lack of nationwide real-world studies estimating life expectancy (LE), loss-of-LE, life-years saved, and lifetime medical costs. We evaluated the cost-effectiveness of PCI versus non-PCI therapy by integrating a survival function and mean-cost function over a lifelong horizon to obtain the estimations for AMI patients without major comorbidities. We constructed a longitudinal AMI cohort based on the claim database of Taiwan's National Health Insurance during 1999–2015. Taiwan's National Mortality Registry Database was linked to derive a survival function to estimate LE, loss-of-LE, life-years saved, and lifetime medical costs in both therapies. This study enrolled a total of 38,441 AMI patients; AMI patients receiving PCI showed a fewer loss-of-LE (3.6 versus 5.2 years), and more lifetime medical costs (US$ 49,112 versus US$ 43,532). The incremental cost-effectiveness ratio (ICER) was US$ 3488 per life-year saved. After stratification by age, the AMI patients aged 50–59 years receiving PCI was shown to be cost-saving. From the perspective of Taiwan's National Health Insurance, PCI is cost-effective in AMI patients without major comorbidities. Notably, for patients aged 50–59 years, PCI is cost-saving.

## Introduction

Acute myocardial infarction (AMI), including ST-segment elevation myocardial infarction (STEMI) and non-ST-segment elevation myocardial infarction (NSTEMI), is one of the major leading causes of mortality in developed countries^1^. Reperfusion therapy, consisting of percutaneous coronary intervention (PCI) and non-PCI therapy, has been widely used for AMI. Patients with STEMI, nowadays, are suggested to receive reperfusion therapy as soon as possible, either thrombolytic therapy or primary PCI which is defined as coronary intervention performed in the occluded artery without any previous thrombolysis^2–5^. Among these therapies, a meta-analysis including 23 randomized trials further showed that patients with STEMI receive more clinical benefits from primary PCI than thrombolytic therapy alone in terms of overall mortality, cardiovascular death, repeated myocardial infarction, and stroke^6^. Although PCI is not recommended for all NSTEMI patients, these patients seem to show better outcomes after early PCI, if they belong to high-risk groups with more unstable conditions^7–10^.

With the development of advanced PCI and guideline-directed medicine, several studies have shown that the incidence and mortality of AMI in Western countries is decreasing^11–13^. However, in Taiwan, the incidence of AMI continues to increase from 49.8 per 100,000 persons in 2009 to 50.7 per 100,000 persons in 2015, and the in-hospital mortality of NSTEMI has not mitigated significantly^14^. Among the findings, especially younger populations (aged < 55 years), seemed to show a significantly increased proportion of NSTEMI, i.e., 30.3% for males and 29.4% for females^14^.

In general, most previous studies merely estimated annual medical costs, instead of a comprehensive projection of lifetime medical expenditure^15–17^. With an increasing burden of AMI, rising medical costs, and limited healthcare resources, there is a need to assess the impacts and cost-effectiveness of PCI versus non-PCI therapy on life expectancy (LE), loss-of-LE, life-years saved and lifetime medical costs, to improve the sustainability of the universal coverage system. Therefore, this study aimed to use a population-based cohort followed for 18 years to evaluate the impact of PCI versus non-PCI therapy on cost-effectiveness among patients with AMI.

## Methods

### Data and sample

Taiwan has a single-payer and universal-coverage national healthcare system^18^. The National Health Insurance (NHI) was launched in 1995, and it covers over 99% of the 23 million residents. This system also deals with the financing of healthcare and reimbursement of all medical claims^19^. This study was commenced after approval by the Institutional Review Committee of National Cheng Kung University Hospital (NCKUH, B-ER-105-386), involving the interlinkage of databases of the NHI and National Mortality Registry from 1999 to 2016.

To establish the AMI cohort, we identified subjects from the NHI research database by the following criteria: first, we defined AMI patients as those admitted to hospital due to AMI [International Classification of Diseases, Ninth Revision, Clinical Modification (ICD-9-CM) 410.xx] in any year during 1998–2015; and aged between 20 and 99 years (n = 200,491). Second, to clarify first-time in-patient AMI, we excluded those who had a diagnosis of AMI in 1998, those who had the ICD-9 code of 412 in the same admission, and those who had missing data on age, gender, or prior outpatient or emergency department records before their admission (n = 53,859). Third, to control for potential confounding, we also excluded patients who had major comorbidities before AMI hospitalization, including ischemic stroke (ICD-9-CM code: 433, 434, 435, 437, 438), hemorrhagic stroke (ICD-9-CM code: 430, 431, 432), chronic kidney diseases (CKD, ICD-9-CM code: 016.0, 095.4, 189.0, 189.9, 223.0, 236.91, 250.4, 271.4, 274.1, 283.11, 403.X1, 404.X2, 404.X3, 440.1, 442.1, 447.3, 572.4, 580-588, 591, 642.1, 646.2, 753.12-753.17, 753.19, 753.2, 794.4), liver cirrhosis (ICD-9-CM code: 571.2, 571.5, 571.6), chronic obstructive pulmonary diseases (COPD, 491, 492, 493.2, 496), and any documented malignancy (ICD-9-CM code: 140-208)^20^.

To classify whether an AMI patient received PCI or not, we identified the intervention. The therapeutic procedures were defined according to the procedure codes in the NHI database: diagnostic coronary angiography (CAG; ICD-9-CM code: 88.55, 88.56, 88.57), percutaneous transluminal coronary angioplasty (PTCA; ICD-9-CM code: 36.01, 36.02, 36.05, 36.09), coronary artery stenting (ICD-9-CM code: 36.06, 36.07), and coronary artery bypass graft (CABG; ICD-9-CM code: 36.10, 36.11, 36.12, 36.13, 36.14, 36.15, 36.16). Patients receiving PCI therapy were defined as those who received PTCA or PTCA plus coronary artery stenting during hospitalization. For those with non-PCI therapy, we excluded the patients with myocardial infarction with non-obstructive coronary artery (MINOCA) to reduce the heterogeneity between PCI and non-PCI group. MINOCA included coronary spasm (ICD-9-CM code: 413.1), coronary bridge (ICD-9-CM code: 414.8), myocarditis/pericarditis/endocarditis (ICD-9-CM code: 420, 421, 422, 423, 424, 429), cardiomyopathy (ICD-9-CM code: 425), sepsis/shock/non-cardiogenic shock (ICD-9-CM code: 995), and trauma (ICD-9-CM code: 959). We also excluded those who did not take dual anti-platelet agents within 3 months after the AMI diagnosis. Thus, the total number of identified subjects with AMI enrolled in this current study was 38,441.

### Statistical analysis

#### Estimation of life expectancy, loss of life expectancy, and life-year saved

By interlinking the Taiwan National Mortality Registry and the reimbursement database of NHI, we obtained 38,441 subjects with a new-AMI admission from 1999 to 2015. Among them, 26,193 patients received PCI, while 12,248 received non-PCI therapy.

We applied the Kaplan–Meier method to estimate the survival function in the follow-up period of 18 years (namely, 1999–2016) and extrapolated to lifetime by a semiparametric method^21^. Then, we applied Monte–Carlo methods to obtain the lifetime survival function from age-, gender- and calendar year-matched referents simulated from the National Vital Statistics database. With premature mortality caused by AMI, the relative survival between the index cohort and the reference population ranged between 0 and 1. Next, we performed logit transformations of the relative survivals obtained from the above methods, and the curve often decreased quickly right after the occurrence of disease and gradually approached a straight line if the assumption of a constant excess hazard was satisfied^22^. Then, with the linearity property of the logit transformed curve, a restricted cubic splines model was fitted, which was linear beyond the last knot, to the observed curve. We used the fitted model to extrapolate the first month beyond follow-up, which was generally accurate and was treated as a new “observed value”. Month-by-month, we repeatedly updated the data of the logit transformed function by dropping the first value and adding the new “observed value”, and re-fitted the restricted cubic splines model to obtain updated data for predicting values in the following months. When the rolling extrapolation of the logit transformed function was completed, the survival function of the index cohort beyond the maximum follow-up period was then obtained from the back-transformed relative survival function and survival function of the referents (Supplementary Figure S1).

Thereby, we estimated the lifetime survival, and the area under the survival curve which was the LE of the patients with AMI. We subtracted the LE of the AMI patients from the corresponding age-, gender- and calendar year-matched reference population to obtain the loss-of-LE. Also, we generated the standard errors (SE) of life-years saved and loss-of-LE with a bootstrap method, which used 100 times of repeated sampling from real-world datasets to implement the extrapolation process.

#### Estimation of lifetime medical costs

The lifetime medical costs for patients with AMI in this study was defined as direct medical costs since the date of AMI diagnosis to the date of death, but did not include direct non-medical costs, indirect costs, nor long-term care costs. The direct medical costs recorded in the NHI database included inpatient hospitalization, emergent department visits, outpatient clinic visits, medicine prescriptions, surgeries, and invasive therapies. To further analyze the direct medical costs, we divided the lifetime medical costs into the costs for the first AMI hospitalization, and outpatient and inpatient lifetime medical costs after the first AMI.

Based on the global budget policy launched by Taiwan’s NHI, we obtained cost estimations from the NHI database by using quarterly point value tables. Taking into account the increased medical spending near the end-of-life, we applied the rolling extrapolation survival-adjusted cost estimator to obtain more accurate medical costs over lifetime. Besides, a previous study demonstrated that a weighted average of the mean costs of deceased patients and the expected costs of surviving patients in a time interval can represent the mean aggregated medical costs^23^. Hence, we drew a time series plot of the mean spending of patients in the 24 months prior to their death and the mean monthly costs for patients who stayed alive in the 36 months prior to the end of follow-up. The reason is that healthcare spending for AMI was typically found to increase substantially in the 24 months before death. After setting these parameters, lifetime medical costs were estimated by two methods, the values of survival function from the rolling extrapolation algorithm, and the values of the monthly mean cost function from the survival-adjusted cost estimator^21^.

With estimates of LE, loss-of-LE, life-years saved, and lifetime medical costs for the AMI patients with PCI and non-PCI therapy, we calculated the differences in LE, loss-of-LE and lifetime medical costs in both therapies. Subsequently, we derived the ICERs of PCI versus non-PCI among the patients with AMI. All lifetime medical costs were discounted at a rate of 3% and 0% to take into account the factor of time according to the recommended guidelines of methodological standards for pharmacoeconomic evaluations^24^. Moreover, we noted that the medical costs are significantly higher than the average costs several months before death, and the values of month varied according to the different age stratification. Therefore, we performed a further sensitivity analysis with different K values for the uncertain period before death in the different age stratification (Supplementary Tables S4, S5). The current study converted all costs to US dollars (Exchange rate 1 US dollar = 31.92 New Taiwan dollars in 2015).

To estimate the number of life-years saved by intervention and ICER, we would usually apply the following formula for data collected from randomized trials:

$${\text{ICER}}\, = \,\left( {{\text{Lifetime medical costs}}_{{({\text{PCI}})}} - {\text{Lifetime medical costs}}_{{({\text{non}} - {\text{PCI}})}} } \right)/\left( {{\text{LE}}_{{({\text{PCI}})}} - {\text{LE}}_{{({\text{non}} - {\text{PCI}})}} } \right)$$

However, in the real world, the two compared cohorts might come from different age and sex distributions. People who are older, female, and/or co-morbid with other major diseases would be less likely to receive PCI, which is also a health disparity. Hence, we adjusted for different distributions of sex, age, and calendar-year of diagnosis to obtain a more accurate estimation of life-years saved by intervention. Namely, we decided to use the difference in loss-of-LE instead of LE, modified as follows:

$${\text{Formula}}:\left( {{\text{Lifetime medical costs}}_{{({\text{PCI}})}} - {\text{Lifetime medical costs}}_{{({\text{non}} - {\text{PCI}})}} } \right)/\left( {{\text{Loss}} - {\text{of}} - {\text{LE}}_{{({\text{non}} - {\text{PCI}})}} - {\text{Loss}} - {\text{of}} - {\text{LE}}_{{({\text{PCI}})}} } \right)$$

In addition, we used this longitudinal cohort data to determine the incidence of adverse cardiovascular events, including repeated myocardial infarction, stroke, and heart failure, within different follow-up periods, i.e., 6 months, 1 year, 3 years and lifetime or to the end of the 18th year.

We performed descriptive analyses where the continuous variables are presented as means and standard deviations, and dichotomized variables are presented as percentages. The differences between each group was tested by t test for continuous variables and chi-square test for categorical variables. Descriptive statistical analyses and survival analyses were performed using the SAS software V9.4 (SAS Institute Inc.). Besides, we conducted the analyses for the rolling extrapolation to estimate LE, loss-of-LE, and lifetime medical costs by using the iSQoL2 software.

## Results

**Figure 1:**
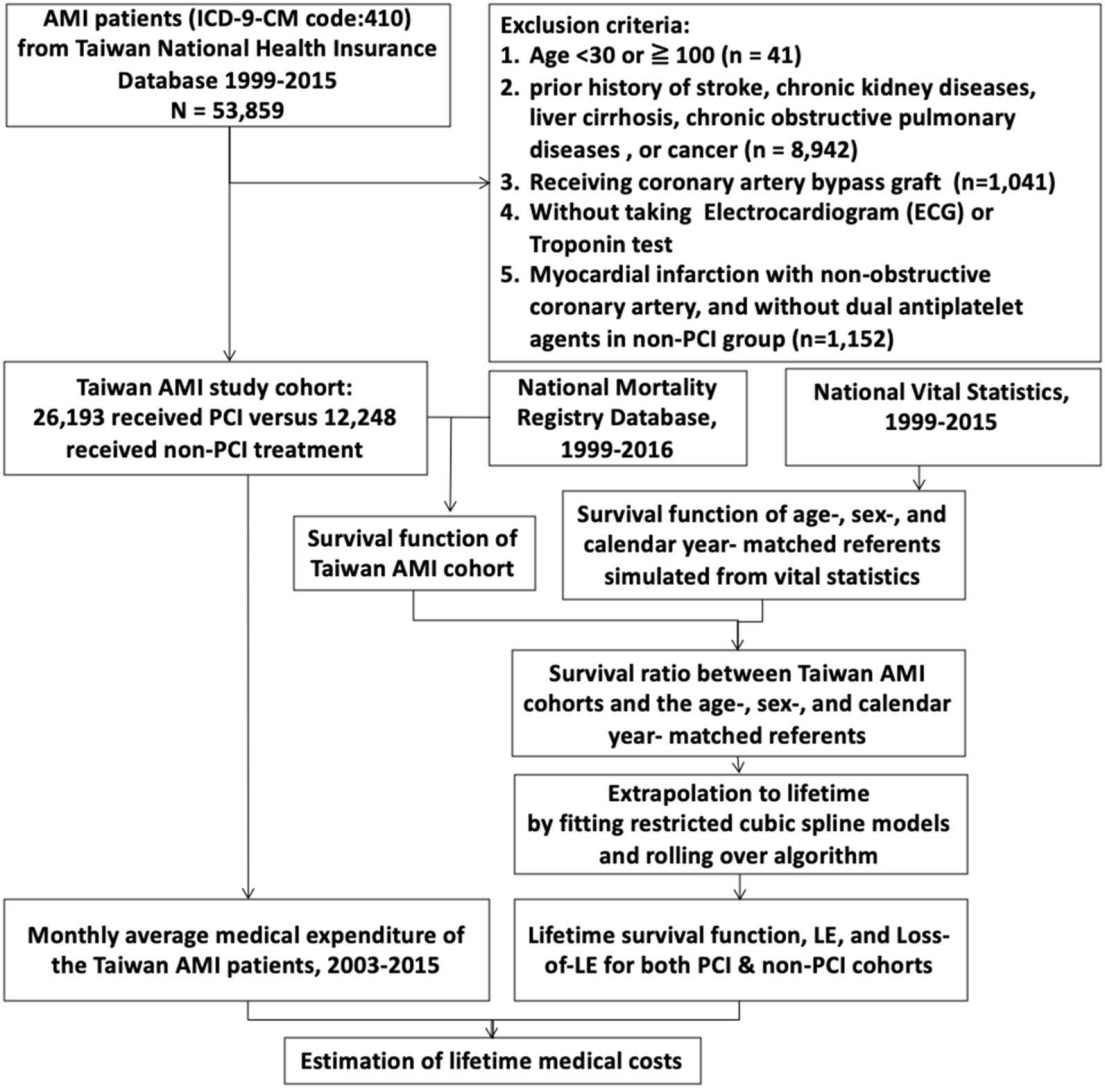
Flow diagram of cohort selection and estimation for lifetime medical costs. *AMI* acute myocardial infarction, *ICD-9-CM* International Classification of Diseases, Ninth Revision, Clinical Modification, *PCI* percutaneous coronary intervention, *LE* life expectancy, *Loss-of-LE* loss of life expectancy.

Figure 1 presents the flow diagram of the enrollment process of AMI patients in the Taiwan NHI database, and the estimations of lifetime survival function, LE, loss-of-LE, life-years saved, and lifetime medical costs. The total number of patients with AMI during 1999 and 2015 was 38,441 subjects without comorbidities, including 26,193 in PCI group and 12,248 in non-PCI one. In Table 1, the group without comorbidities, about two-thirds received PCI, while in the group with comorbidities, slightly less than half received PCI. Male patients made up a higher percentage in the PCI group than the non-PCI group regardless of comorbidities. The proportion of STEMI in PCI group is smaller than that in non-PCI group (16.4% versus 23.4%), while NSTEMI in PCI group is higher than non-PCI group (83.6% versus 76.6%). In the group without major comorbidities, the mean age of those receiving PCI was 4.0 years younger than those receiving non-PCI therapy, while those with comorbidities were about 8–10 years older than those without comorbidities. The direct medical cost for the first hospitalization in PCI group (US$ 5486) was higher than non-PCI group (US$ 4728). Likewise, the follow-up outpatient and inpatient lifetime medical costs in PCI group was more than those in non-PCI, i.e., US$ 23,975 versus US$18,860 and US$36,286 versus US$29,823 (details in Supplementary Table S1).

**Table 1:** Demographic characteristics of patients with acute myocardial infarction who later developed adverse cardiovascular events (ACE) and direct medical costs for the first hospitalization, life-time medical costs (LMC) for in- and out-patient stratified by major comorbidities and receiving percutaneous coronary intervention (PCI).

Receiving PCI	Without major comorbidities		With major comorbidities	
	Yes	No	Yes	No
Total	26,193	12,248	4369	4573
No. of male (%)	21,675 (82.8%)	9202 (75.1%)	3238 (74.1%)	3109 (68.0%)
Age (mean ± SD)	60.9 ± 13.4	64.9 ± 13.7	70.3 ± 11.9	72.8 ± 12.1
No. of STEMI (%)	4306 (16.4%)	2863 (23.4%)	N/A	N/A
No. of NSTEMI (%)	21,887 (83.6%)	9385 (76.6%)	N/A	N/A
No. of deceased (%)	5802 (22.2%)	5397 (44.1%)	2382 (54.5%)	3504 (76.6%)
Direct medical costs for the first hospitalization (US$)	5486	4728	N/A	N/A
Follow-up outpatient LMC (US$)	23,975	18,860	N/A	N/A
Follow-up inpatient LMC (US$)	36,286	29,823	N/A	N/A
**ACE (No. events/population)**				
Within 1 year				
Acute myocardial infarction	1317 (5.0%)	1529 (12.5%)	322 (7.4%)	597 (13.1%)
Heart Failure	753 (2.9%)	499 (4.1%)	300 (6.9%)	363 (7.9%)
Stroke	465 (1.8%)	271 (2.2%)	171 (3.9%)	163 (3.6%)
Within 3 years				
Acute myocardial infarction	1739 (6.7%)	1751 (14.3%)	403 (9.2%)	688 (15.0%)
Heart Failure	1096 (3.5%)	688 (5.6%)	400 (9.2%)	493 (10.8%)
Stroke	831 (2.6%)	510 (4.2%)	279 (6.4%)	245 (5.4%)
Within 18 years				
Acute myocardial infarction	2749 (10.5%)	2247 (18.3%)	517 (11.8%)	783 (17.1%)
Heart Failure	1732 (6.6%)	1117 (9.1%)	510 (11.7%)	602 (13.2%)
Stroke	1652 (6.3%)	983 (8.0%)	404 (9.2%)	323 (7.1%)

Major comorbidities include ischemic stroke, hemorrhagic stroke, chronic kidney diseases, liver cirrhosis, chronic obstructive pulmonary diseases, and documental malignancy.

*SD* standard deviation, *STEMI* ST-segment elevation myocardial infarction, *NSTEMI* non ST-segment elevation myocardial infarction.

Table 1 also shows a consistent trend in AMI patients without comorbidities receiving PCI who developed lower incidence rates of adverse cardiovascular events, i.e., AMI, heart failure, and stroke, within different follow-up periods. Additionally, those receiving PCI in the group with major comorbidities developed lower incidences of acute MI and heart failure compared to those receiving non-PCI therapy regardless of the length of follow-up periods, but the occurrences of stroke did not show the same trend. Furthermore, the total AMI population with/without comorbidities stratified by age in 10-years age groups are presented in Supplementary Table S2.

**Figure 2:**
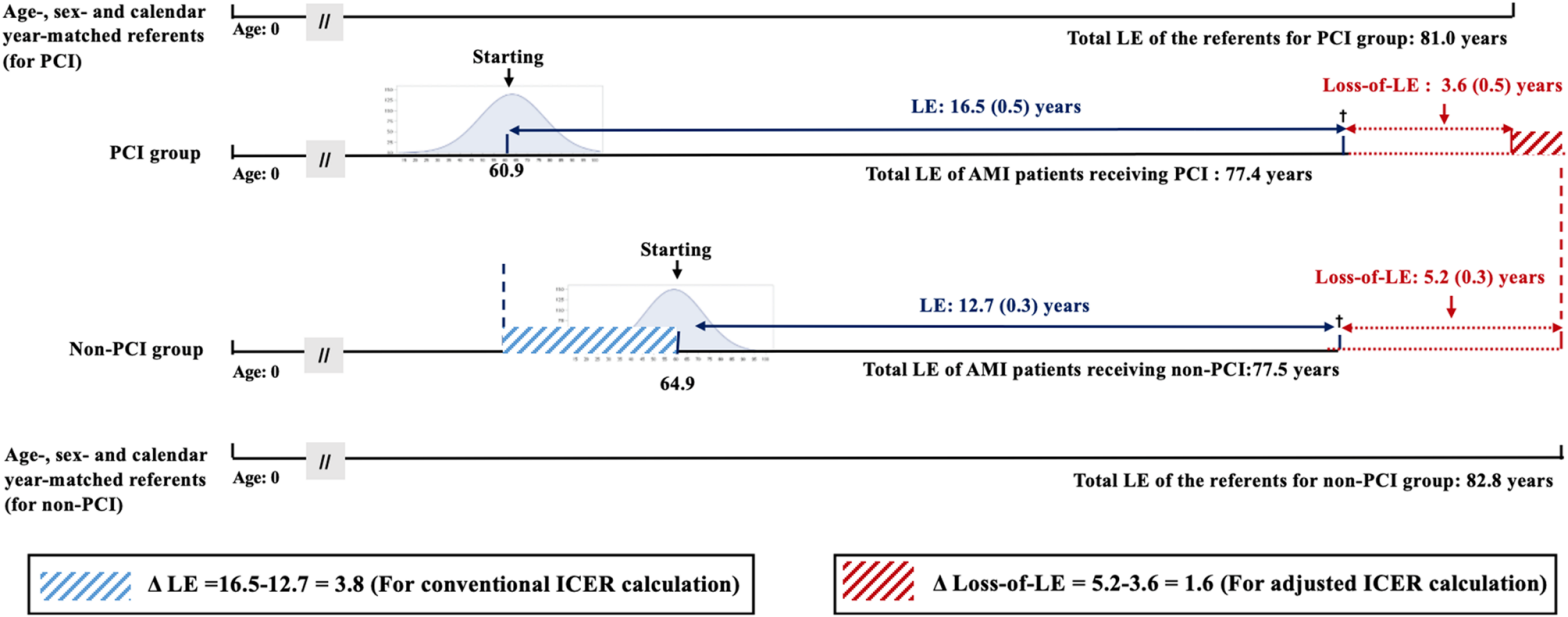
Illustration of the estimation of life expectancy (LE), total LE, loss of life expectancy (Loss-of-LE), and life-year saved for patients with acute myocardial infarction (AMI) receiving percutaneous coronary intervention (PCI) and non-PCI, and their corresponding referents matched with same age-, sex-, and calendar year of diagnosis. It indicates that comparison of loss-of-LE to obtain difference-in-differences as the lifetime health benefits for PCI would adjust for different distributions of age, sex, and different medical technology offered in different calendar years between the two cohorts.

Figure 2 illustrates the LE, loss-of-LE, and life-years saved for AMI patients without major comorbidities. LE after an AMI episode in the PCI and non-PCI group were 16.5 and 12.7 years, respectively, while those of the age-, gender- and calendar year-matched population in the PCI and non-PCI group were 81.0 and 82.8 years. Hence, the loss-of-LE in the PCI and non-PCI groups were 3.6 [= 81.0 − (60.9 + 16.5)] and 5.2 [= 82.8 − (64.9 + 12.7)] years, respectively, and the difference-in-differences of loss-of-LE of both therapies would be 1.6 years. In other words, Fig. 2 shows how different distributions of age, sex, and calendar year between the two different cohorts of PCI and non-PCI could be adjusted by comparing the loss-of-LE instead of LE. As the calendar year of diagnosis was matched, it accounts for different stages of medical technology in different periods.

Table 2 summarizes the results of LE, loss-of-LE, lifetime medical costs, and ICERs for PCI stratified by age groups, which were adjusted to the year 2015 with a discount rate of 3%. The overall ICER of PCI versus non-PCI therapy calculated by the difference in LE and loss-of-LE were US$1468 and US$3488 per life-year saved, respectively. As the mean age of the PCI cohort was younger than that of the non-PCI cohort, the conventional ICER values by LE (US$ 1468 per life-year saved) could have been over-estimated. We calculated the difference-in-differences by comparing the loss-of-LE, which resulted in an adjusted ICER (US$ 3488 per life-year saved). Among them, the groups aged 50–59 years were shown to be cost-saving when considered from a lifetime horizon.

**Table 2:** Life expectancy (LE), loss of life expectancy (Loss-of-LE), life-year saved, lifetime medical cost (LMC, adjusted to year 2015 with a discount rate of 3%) and incremental cost-effectiveness ratio (ICER) of patients with acute myocardial infarction stratified by receiving percutaneous coronary intervention (PCI).

Age (years)	PCI			Non-PCI			PCI versus non-PCI			
	LE	Loss-of-LE	LMC (US$)	LE	Loss-of-LE	LMC (US$)	∆LE	∆Loss-of-LE	Conventional ICER (US$/life-year saved)	Adjusted ICER (US$/life-year saved)
**Discount rate = 3%**										
30–99	16.5 (0.5)	3.6 (0.5)	49,112	12.7 (0.3)	5.2 (0.3)	43,532	3.8	1.6	1468	3488
30–39	27.1 (2.7)	6.8 (2.7)	55,206	21.0 (3.0)	12.4 (3.0)	40,523	6.1	5.6	2407	2622
40–49	22.8 (1.7)	6.4 (1.7)	54,538	20.6 (1.3)	8.2 (1.3)	51,329	2.2	1.8	1459	1783
50–59	20.5 (1.0)	3.7 (1.0)	56,847	19.0 (0.6)	4.9 (0.6)	60,613	1.5	1.2	-2511	-3138
60–69	15.0 (0.4)	3.3 (0.4)	51,723	12.5 (0.3)	5.6 (0.3)	46,311	2.5	2.3	2165	2353
70–79	10.2 (0.2)	1.9 (0.2)	40,924	7.2 (0.2)	4.7 (0.2)	35,146	3.0	2.8	1926	2064
80–99	5.7 (0.2)	1.2 (0.2)	25,038	3.3 (0.1)	3.2 (0.1)	21,543	2.4	2.0	1456	1748

∆ LE was derived from (LE in PCI–LE in non-PCI).

∆ Loss-of-LE was derived from (Loss-of-LE in non-PCI–Loss-of-LE in PCI).

## Discussions

This study is the first to utilize long-term survival data to quantify LE, loss-of-LE, life-years saved, lifetime medical costs, and ICER of PCI versus non-PCI therapy in AMI patients without major comorbidities. Although a previous study projected the cost-effectiveness of PCI through modeling^25^, the current study provided an alternative way to corroborate or validate the prediction by using a real-world database. Since this study is not a randomized clinical trial, our results of the health benefits from PCI cannot be directly considered as causal. However, we have the following arguments to corroborate the above hypothesis: first, since we excluded all AMI patients with major comorbidities from the beginning, the health benefits cannot be attributed to potential confounding by the presence of other diseases, i.e., malignancy, stroke, COPD, CKD, and liver cirrhosis. Moreover, we also excluded all cases of MINOCA from the non-PCI cohort, which would make the comparison more valid. Second, the ICER calculated by life-years saved was the difference in loss-of-LE (Fig. 2), which was adjusted for different distributions of age, sex, and calendar-year of diagnosis. All the above factors, including the advancement of medical technology in different calendar years, cannot be used to explain the more desirable results of the PCI cohort. Third, as the validation of our extrapolation algorithm (month-by-month, rolling-over from the end of the 9th year to the 18th year) is accurate (as shown in Supplementary Figure S1), we anticipate that the same method using 18 years of real data (with a censored rate of 33%) extrapolated to lifetime would be reasonably accurate^21,22^. Fourth, in addition by adjusting medical expenditures to the different CPI (consumer price index) of each calendar year, we accounted for the annual discount rate and increased spending near the end of life when extrapolating after follow-up^21^. Finally, the patients receiving PCI consistently showed a lower incidence rate of adverse cardiovascular events during the 18 years of follow-up (Table 1), indicating a successful rescue of nearby ischemic myocardium and corroborating the pathophysiologic plausibility of PCI. We thus tentatively conclude that the health benefits of AMI patients receiving PCI could not be attributed to any other alternative explanations.

By inter-linking the NHI database and National Mortality Registry plus borrowing data from National Vital Statistics, this study demonstrated that AMI patients receiving PCI required higher lifetime medical costs (US$49,112 versus US$43,532 of non-PCI). However, AMI patients receiving PCI simultaneously had a longer LE (16.5 versus 12.7 years) and smaller loss-of-LE (3.6 versus 5.2 years). The overall ICER of PCI versus non-PCI in all age groups was a mere US$1468 per life-year saved, which turned out to be US$3488 after adjustment of different age, sex, and calendar-year of diagnosis. Given that the GDP per capita in Taiwan was around US$ 24,000–25,792 during 2013 and 2018^26^, it seems that PCI therapy in AMI patients would be cost-effective, regardless of their age, if we adopt the threshold for cost-effectiveness proposed by the WHO which is one to three times the GDP per capita^27,28^. On the other hand, as there was substantial increase in the proportion of NSTEMI in younger populations (age < 55 years)^14^, the financial benefit may encourage physicians to perform PCI in younger patients when considered from the national payer’s perspective.

We found that the AMI patients aged 50–59 years without major comorbidities consumed the highest lifetime medical costs irrespective of PCI treatment in Taiwan setting. Unlike the other ages, PCI group in this stratum produced fewer lifetime medical costs than non-PCI group, which contributed to a cost-saving result. This would be that PCI may have a direct symptom relief and fewer later complications, which may improve motivation of medical adherence, lifestyle modification and secondary prevention of cardiovascular diseases^29^.

In the sensitivity analyses which accounted for the factor of time, PCI in AMI patients remained very cost-effective, and even cost-saving for those aged 50–59 years, given the discount rate of zero (Supplementary Table S3). Besides, the different period of months (K value) before death according to patients’ age stratification was used to estimate the discrepancy of life-time medical costs (Supplementary Tables S4, S5). The result did not change the economic benefit of PCI versus non-PCI, which may strengthen the robustness of the cost-effectiveness of PCI therapy for AMI patients. Furthermore, Table 1 shows that younger populations and those without major comorbidities seem to prefer PCI more, which is compatible with a previous study^30^. However, in older groups without major comorbidities, we found that PCI still produces a longer LE, smaller loss-of-LE, and better cost-effectiveness (US$2064 and US$1748 per life-year saved in those aged 70–79 and 80–99 years, respectively). These findings may support the performance of PCI in AMI patients throughout all age groups without major comorbidities.

In the past two decades, there has been growing momentum for a paradigm shift from pay-for-volume to pay-for-value^31,32^. Also, the value should be assessed from the perspectives of patients and the society. This issue is particularly challenging when tackled from the aspect of cost-effectiveness from a lifetime horizon. Although PCI may save lives, there is still a potential risk of morbidity and even mortality, especially among older people. Furthermore, for patients, the concern of co-payment and/or out-of-pocket expense resulting from PCI and relevant novel treatments has almost always existed as new technology evolves, which might lead to health disparity, even for those living in a country with universal coverage. With nation-wide data and innovative methods to estimate lifetime survival functions and costs, our study provides a viable solution and evidence on the cost-effectiveness of PCI for AMI patients, including the elderly (Table 2).

This study has the following limitations. First, only STEMI and high-risk NSTEMI patients are suggested to receive PCI^7–9^. The ICER values in high- and low-risk NSTEMI patients may differ. With a lack of actual laboratory data, like ECG patterns, and magnitudes of the rise in biomarkers of myocardial infarction, we were unable to assess the risks in NSTEMI patients accurately. Future studies in different settings are warranted to assess the different risks and comorbidities of AMI patients. Second, Taiwan’s NHI does not comprehensively cover novel treatments and medications associated with PCI, like drug-eluting stents, which are partially paid by the patients themselves. Also, the indirect medical costs, e.g., co-payment and out-of-pocket expense, were not recorded in the reimbursement database. The lifetime medical costs extracted from the NHI database may lead to an underestimation of PCI treatment. However, since most hospitalization costs, including those of intensive care units, were covered, the discrepancy would mainly result from the costs of different types of stents. Future studies are warranted to account for and minimize this potential disparity in cost. Last, using the incremental cost per life-year saved instead of cost per quality-adjusted life-year gained may impact the financial benefits. However, Table 1 shows that AMI patients receiving PCI had a lower incidence of adverse cardiovascular events in the 18-years of follow-up. Namely, those receiving PCI therapy would less likely suffer from later cardiovascular events and have a better quality of life, which was compatible with previous studies^33–35^. Therefore, in consideration of quality-adjusted life-years, PCI therapy would still have more economic incentives for AMI patients.

## Conclusions

This study is the first to utilize real-world, nation-wide, long-term survival data to extrapolate LE, loss-of-LE, and lifetime medical costs for AMI patients without major comorbidities. Our analyses demonstrate the cost-effectiveness of PCI in contrast to non-PCI therapy for AMI patients without major comorbidities, with an ICER of US$3488 per life-year saved; notably, PCI was shown to be cost-effective across different ages and even cost-saving in those aged 50–59 years in Taiwan. More comprehensive studies are needed to consider the values from a societal perspective, which would include productivity loss, social services, quality of life, etc., and stratification by different risks and/or comorbidities.

## Supplementary Information

Supplementary Information
