# Supplementary material for: Cost-effectiveness of percutaneous coronary intervention versus medical therapy in patients with acute myocardial infarction: real-world and lifetime-horizon data from Taiwan
Source: Sci Rep. 2021 Mar 10;11:5608. doi: 10.1038/s41598-021-84853-y (PMC7947011; doi:10.1038/s41598-021-84853-y)
Supplement: Supplementary file 1 — Supplementary Information [file 41598_2021_84853_MOESM1_ESM.docx]

**Cost-effectiveness of percutaneous coronary intervention versus medical therapy in patients with acute myocardial infarction: real-world and lifetime-horizon data from Taiwan**

Chia-Te Liao MD, M.Sc.^1,2,3^, Tung-Han Hsieh MD^4^, Chia-Yin Shih MS^2^, Ping-Yen Liu MD, PhD^4^, Jung-Der Wang MD, Sc.D.^2*^

^1^ Division of Cardiology, Department of Internal Medicine, Chi-Mei Medical Center, Taiwan

^2^ Department of Public Health, College of Medicine, National Cheng Kung University; Departments of Internal Medicine and Occupational and Environmental Medicine, National Cheng Kung University Hospital Tainan, Taiwan

^3^ Department of Electrical Engineer, Southern Taiwan University of Science and. Technology, Tainan, Taiwan

^4^ Division of Cardiology, Department of Internal Medicine, National Cheng Kung University Hospital, Taiwan

Word counts: 3477

***Corresponding author**

Jung-Der Wang, M.D., Sc.D.

Department of Public Health, College of Medicine, National Cheng Kung University; Departments of Internal Medicine and Occupational and Environmental Medicine, National Cheng Kung University Hospital Tainan, Taiwan

Address: No.1, University Road, Tainan, TAIWAN 701

Tel: +886-6-2353535 ext 5600; Fax: +886-6-2359033

Email: jdwang121@gmail.com

| **Supplementary** Table 1. Direct medical costs of patients with acute myocardial infarction (AMI) who receive percutaneous coronary intervention (PCI) and non-PCI are stratified by first AMI hospitalization, outpatient and inpatient lifetime medical costs. | | | |
| --- | --- | --- | --- |
| Discount rate = 3% | | | |
|  | **PCI** |  | **Non-PCI** |
| First hospitalization (US$) | | | |
| Mean **±** standard deviation | 5,486 **±** 3,104 |  | 4,728 **±** 7,443 |
| Median | 4,816 |  | 2,346 |
| Min | 378 |  | 30 |
| Max | 121,305 |  | 153,860 |
| Follow-up outpatient lifetime medical costs (US$) | 23,975 |  | 18,860 |
| Follow-up inpatient lifetime medical costs (US$) | 36,286 |  | 29,823 |

| **Supplement** Table 2. Different age groups of patients with acute myocardial infarction stratified by major comorbidities and receiving percutaneous coronary intervention (PCI). | | | | |
| --- | --- | --- | --- | --- |
|  | Without major comorbidities | | With major comorbidities | |
| Receiving PCI | Yes | No | Yes | No |
| Total numbers | 26,193 | 12,248 | 4,369 | 4,573 |
| 30-39 years | 1,217 (4.6%) | 468 (3.8%) | 43 (1.0%) | 35 (0.8%) |
| 40-49 years | 4,393 (16.8%) | 1,589 (13.0%) | 189 (4.3%) | 155 (3.4%) |
| 50-59 years | 7,114 (27.2%) | 2,632 (21.5%) | 605 (13.8%) | 501 (11.0%) |
| 60-69 years | 6,103 (23.2%) | 2,638 (21.5%) | 1,056 (24.2%) | 928 (20.3%) |
| 70-79 years | 4,729 (18.1%) | 2,683 (21.9%) | 1,405 (32.2%) | 1,399 (30.6%) |
| 80-99 years | 2,637 (10.1%) | 2,238 (18.3%) | 1,071 (24.5%) | 1,555 (34.0%) |
| Major comorbidities include ischemic stroke, hemorrhagic stroke, chronic kidney diseases, liver cirrhosis, chronic obstructive pulmonary diseases, and documental malignancy. SD, standard deviation | | | | |

| **Supplementary** Table 3. Life expectancy (LE), loss of life expectancy (Loss-of-LE), life-year saved, lifetime medical cost (LMC, adjusted to year 2015 with a discount rate of 0%) and incremental cost-effectiveness ratio (ICER) of patients with acute myocardial infarction stratified by receiving percutaneous coronary intervention (PCI). | | | | | | | | | | | | |
| --- | --- | --- | --- | --- | --- | --- | --- | --- | --- | --- | --- | --- |
| **Discount rate = 0%** | | | | | | | | | | | | |
|  | **PCI** | | |  | **Non-PCI** | | |  | **PCI versus non-PCI** | | | |
| **Age(years)** | LE | Loss-of-LE | LMC  (US$) |  | LE | Loss-of-LE | LMC (US$) |  | ∆LE | ∆Loss-of-LE | Conventional ICER (US$ / life-year saved) | Adjusted ICER  (US$ / life-year saved) |
| **30-99** | 17.4 (0.7) | 4.8 (0.7) | 54,621 |  | 13.7 (0.4) | 6.2 (0.4) | 46,879 |  | 3.7 | 1.4 | 2,092 | 5,530 |
| **30-39** | 32.0 (4.7) | 11.2 (4.7) | 64,845 |  | 22.8 (5.4) | 21.1 (5.4) | 44,266 |  | 9.2 | 9.9 | 2,237 | 2,079 |
| **40-49** | 24.7 (2.6) | 9.6 (2.6) | 58,808 |  | 22.7 (2.1) | 12.0 (2.1) | 58,205 |  | 2.0 | 2.4 | 302 | 251 |
| **50-59** | 21.6 (1.4) | 4.9 (1.4) | 60,095 |  | 20.9 (0.8) | 5.8 (0.8) | 68,038 |  | 0.7 | 0.9 | -11,347 | -8,826 |
| **60-69** | 15.3 (0.5) | 3.8 (0.5) | 52,651 |  | 12.9 (0.4) | 6.2 (0.4) | 47,769 |  | 2.4 | 2.4 | 2,034 | 2,034 |
| **70-79** | 10.3 (0.2) | 1.9 (0.2) | 41,154 |  | 7.2 (0.2) | 4.8 (0.2) | 35,412 |  | 3.1 | 2.9 | 1,852 | 1,980 |
| **80-99** | 5.7 (0.2) | 1.2 (0.2) | 25,050 |  | 3.3 (0.1) | 3.2 (0.1) | 21,558 |  | 2.4 | 2.0 | 1,455 | 1,746 |
| ∆ LE derived from (LE of PCI - LE of non-PCI), used for conventional ICER calculation.  ∆ Loss-of-LE derived from difference-in-differences (Loss-of-LE in non-PCI – Loss-of-LE in PCI), used for adjusted ICER calculation. | | | | | | | | | | | |  |

| **Supplementary** Table 4. Sensitivity analysis for estimating lifetime medical cost (LMC, adjusted to year 2015 with a discount rate of 3%) of patients with acute myocardial infarction stratified by receiving percutaneous coronary intervention (PCI). | | | | | |
| --- | --- | --- | --- | --- | --- |
| Discount rate = 3% | | | | | |
| K value*  Age(years) | **PCI** | |  | **Non-PCI** | |
|  | Determined by age | Fixed at 15 |  | Determined by age | Fixed at 15 |
| 30-39 | 55,206 | 54,621 |  | 43,532 | 43,813 |
| 40-49 | 54,538 | 64,845 |  | 40,523 | 39,242 |
| 50-59 | 56,847 | 58,808 |  | 51,329 | 54,296 |
| 60-69 | 51,723 | 60,095 |  | 46,311 | 47,679 |
| 70-79 | 40,924 | 52,651 |  | 35,146 | 35,156 |
| 80-99 | 52,302 | 41,154 |  | 21,543 | 21,949 |
| *K value: The average cost (C_K_) at time K months prior to death is significantly higher than the average cost (C_K_^+^) of subjects who will not die in K months. | | | | | |

| **Supplementary** Table 5. Sensitivity analysis with different K values for the cost-effectiveness of PCI versus non-PCI among patients experiencing acute myocardial infarction | | | | | |
| --- | --- | --- | --- | --- | --- |
| Discount rate = 3% | | | | | |
| K value* | Determined by age | |  | Fixed at 15 | |
| Methods of estimation | Input the data of whole population in ISQOL2 | Weighted average from age-stratified cohorts |  | Input the data of whole population in ISQOL2 | Weighted average from age-stratified cohorts |
| **PCI versus non-PCI** |  |  |  |  |  |
| ∆Loss-of-LE (years) | 1.6 | 1.8 |  | 1.4 | 1.8 |
| ∆Cost (US$) | 5,580 | 6,270 |  | 8,668 | 16,623 |
| Conventional ICER  (US$ / life-year saved) | 1,468 | 1,650 |  | 2,281 | 4,374 |
| Adjusted ICER  (US$ / life-year saved) | 3,488 | 3,514 |  | 6,191 | 9,318 |
| *K value: The average cost (C_K_) at time K months prior to death is significantly higher than the average cost (C_K_^+^) of subjects who will not die in K months.  PCI: percutaneous coronary intervention. LE: life expectancy. ICER: incremental cost-effectiveness ratio. | | | | | |

**Supplementary Figure 1**. This figure demonstrates the validation of survival function by comparing results of extrapolation from the end of the 9^th^ year to the 18^th^ year and the actual survival rates based on Kaplan-Meier’s (K-M) estimate throughout 18 years of follow-up. The relative bias assuming the K-M estimate as the gold standard was less than 1%.


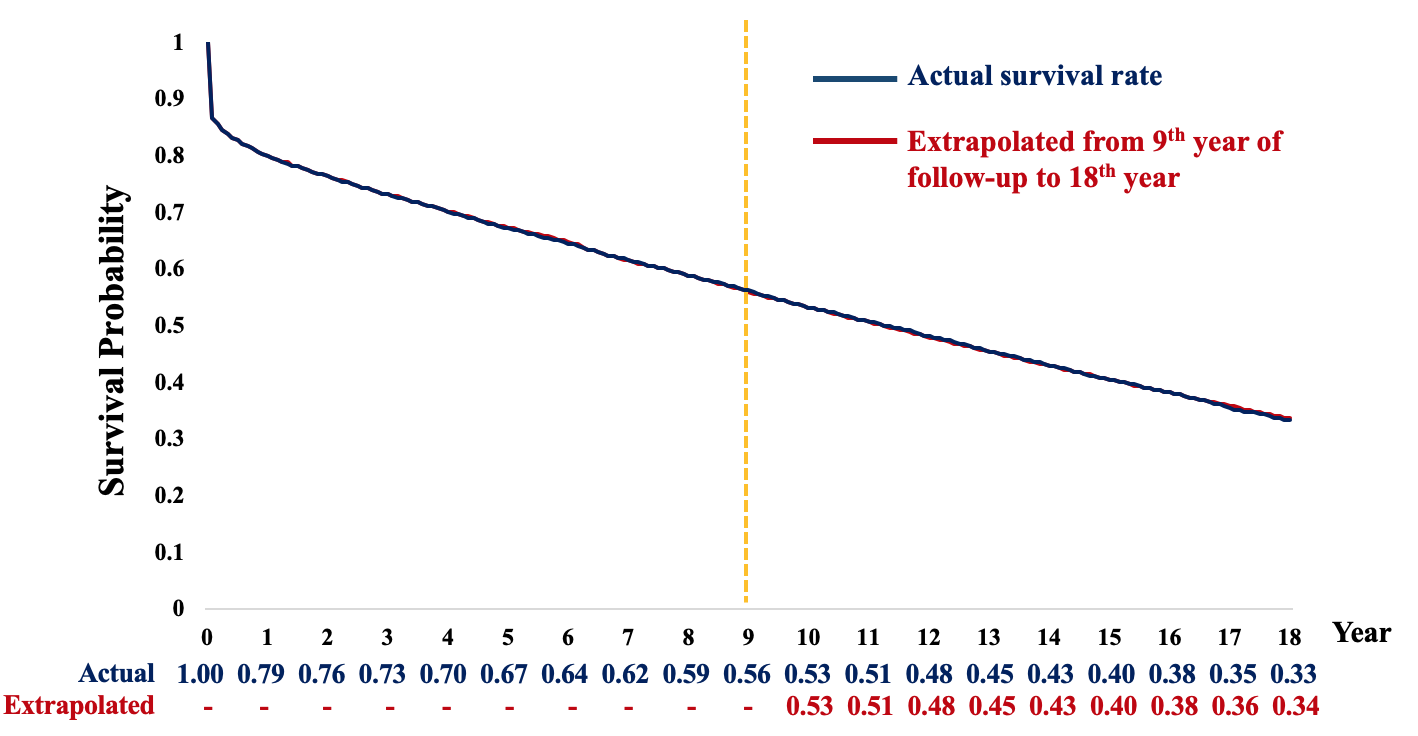


Figure1
